# Smart distribution network voltage estimation using PMU technology considering zero injection constraints

**DOI:** 10.1371/journal.pone.0293616

**Published:** 2024-03-25

**Authors:** Swathi Tangi, D. N. Gaonkar, Ramakrishna S. S. Nuvvula, Polamarasetty P. Kumar, Ilhami Colak, Ahmad F. Tazay, Mohamed I. Mosaad

**Affiliations:** 1 Department of Electrical and Electronics Engineering, Manipal Instiute of Technology (MIT), Manipal Academy of Higher Education (MAHE), Manipal, India; 2 Department of Electrical and Electronics Engineering, National Institute of Technology Karnataka (NITK), Surathkal, India; 3 Deparmtent of Electrical and Electronics Engineering NMAM Institute of Technology, Nitte, Karkala, Karnataka, India; 4 Department of Electrical and Electronics Engineering, GMR Institute of Technology, Rajam, India; 5 Department of Electrical and Electronics Engineering, Faculty of Engineering and Architecture, Nisantasi University, Istanbul, Turkey; 6 Electrical Engineering Department, Colleague of Engineering, Al Baha University, Al Baha, KSA; 7 Electrical & Electronics Engineering Technology Department, Yanbu Industrial College (YIC), Royal Commission Yanbu Colleges & Institutes, Yanbu, Saudi Arabia; Vellore Institute of Technology, INDIA

## Abstract

To properly control the network of the power system and ensure its protection, Phasor measurement units (PMUs) must be used to monitor the network’s operation. PMUs can provide synchronized real-time measurements. These measurements can be used for state estimation, fault detection and diagnosis, and other grid control applications. Conventional state estimation methods use weighting factors to balance the different types of measurements, and zero injection measurements can lead to large weighting factors that can introduce computational errors. The offered methods are designed to ensure that these zero injection criteria can be strictly satisfied while calculating the voltage profile and observability of the various distribution networks without sacrificing computing efficiency. The proposed method’s viability is assessed using standard IEEE distribution networks. MATLAB coding is used to simulate the case analyses. Overall, the study provides a valuable contribution to the field of power distribution system monitoring and control by simplifying the process of determining the optimal locations for PMUs in a distribution network and assessing the impact of ZI buses on the voltage profile of the system.

## 1. Introduction

A power system’s synchronized current and voltage phasors can be measured using equipment like phasor measurement units (PMUs). PMU is among the most crucial measuring tools for advanced intelligent power systems. PMUs, which could also continually record samples of the electrical system’s system parameters, have drawn much interest in distribution networks. These examples can be used for real-time security, supervision, and regulation. Due to the scattered varying energy supplies, the distribution system exhibits distinct dynamics (like wind and solar energy). Furthermore, the smart grid paradigm would convert the current unidirectional power distribution system to a bidirectional one. PMUs must always be deployed at the necessary nodes for complete visibility of the voltage or current phasors.

PMUs give network administrators greater visibility and allow them to pick up incidents that SCADA might have missed in [1]. Most high-voltage buses in real power systems are zero-injection buses without load or generation. They ensure that such zero injection conditions can be rigorously met without compromising predictive performance. An effective zero injection limitation computing technique is required to determine the power system’s state in [2]. In observability assessment, zero-injection pertains to the system’s effects or impacts on buses where no power is supplied. The number of PMUs could be decreased by simulating the zero-injection (ZI) buses present in the situation. As a result, fewer PMUs are needed to analyse the network when zero-injection buses are presented in [3]. ZI buses are regarded as a bus with exceptionally effective power monitoring since they have no supply or demand in [4]. Integrating the implications of ZI buses and load flow observations to figure out the right amount of deployed PMUs, which does not just serve to save cost but much more significantly improves the cyber resilience of the power grid, could be challenged in PMU placement optimization [5]. The term "set of ZI nodes" refers to the ZI bus and any associated buses. If there are N buses in this collection, then using the ZI buses’s functionality to make an unobservable bus observable using the measurability of N-1 buses is sufficient [6].

The following studies have addressed the significance of zero injection nodes in the literature: This study proposes an optimal PMU placement (OPP) problem setting with ZI buses in typical system states and considers contingency due to PMU outage [7]. Adding ZI buses will result in lines being removed from the system, influencing the PMU placement problem outcomes that aim to achieve reliability against a line failure in [8]. A comprehensive PMU-observability framework of the network topology is created in [9], built on the OPP paradigm and considering ZI buses. In [10], considers both the price and how significantly the PMU placements will enhance system observability in the existence of potential vulnerabilities. This paper offers zero injection observation depth (ZIOD) to illustrate how ZI impact propagates. By constraining ZIOD, the authenticity of ZI observation is ensured [11]. The study prioritizes ZIB modelling for optimal PMU placement, adding unique observability constraints (H, M, D) in [12]. In observability assessment, zero-injection pertains to the system’s effects or impacts on buses where no power is supplied. The number of PMUs could be decreased by simulating the ZI buses present in the situation. As a result, fewer PMUs are needed to analyse the network when zero-injection buses are present in [3]. ZI buses are regarded as a bus with exceptionally effective power monitoring since they have no supply or demand in [4]. Integrating the implications of ZI buses and load flow observations to figure out the right amount of deployed PMUs, that not just serves to save cost but much more significantly improves the cyber resilience of the power grid, could be challenged in PMU placement optimization [5]. The term "set of ZI nodes" refers to the ZI buses and any associated buses. If there are N buses in this collection, then using the ZI buses’ functionality to make an unobservable bus observable using the measurability of N-1 buses is sufficient [6].

Reference [13] explained the best locations to strategically install a certain number of PMUs are determined for a system that considers uncertainty and topological aspects. The attack-resistant OPP technique proposed in this research uses reinforcement learning guided tree search to arrange PMUs in a specific order, with the exploration of placement orders being done via reinforcement learning’s sequential decision-making [14]. The unique aspect of this work is positioning the PMUs so that weak buses can be observed with the most significant possible number of PMUs to determine the system’s full observability and stability under various conditions, such as taking zero injection nodes into account and experiencing a single PMU outage in [15]. The two distinct stages: in phase 1, the coordination sites in the search tree are examined by depth-first search, and in phase 2, the primary simplex is configured using the basic feasible solution from phase 1 till the global optimum is identified in [16]. The strategy for deploying micro-phasor measuring units in a distribution network that can operate in different configurations is outlined in [17]. This study introduces the idea of observability propagation depth to improve the OPP formulation that effectively addresses constrained quantitative measurements [18]. Concerning the PMU outage contingency, the nonlinearity in state estimation dependability and resiliency issues are addressed in [19]. This work presents an algorithm that can account for realistic requirements such as redundancy in monitoring crucial system components and calculating the tap ratios of the transformers in use [20]. To assign and identify smart grid anomalies and failures, this project aims to create a uniquely dynamic structure that only needs a small number of PMUs to interact in a protected blockchain-enabled framework in [21]. This study offers a novel strategy for allocating μ-PMUs in flexible smart distribution grids considering zero injection nodes and communication system needs [22]. The impacts of zero injection buses and traditional metrics for PMU placement are carefully considered by a novel integrated model reported in this article [23].

Reference [24] considers single-line and PMU fault-related system observability, either with or without uncertainties. Additionally, it focuses on the effects of potential zero-injection nodes, and a preliminary limitation on the quantity and type of PMU calculations carried out at each node. When used with conventional networks, the suggested technique partitions the network into smaller segments with less reliance, rendering it augments [25]. Global optimal solutions are provided, and the OPP problem is expressed in a direct and precise mathematical manner, guaranteeing the positive quantitative measurements of the corresponding gain multipliers. Additionally, they account for any existing typical measurements, zero injections, and the influence of PMU channel constraints in [26]. A homogeneous PMU deployment strategy is put forth in this research while considering the validity of zero injection observation [11]. Reference [27] recommends deploying phasor measurement devices with the best safety placement and optimum observability reliability. The initial investment and measurement accuracy are both used to distinguish the options requiring the same quantity of PMUs to be deployed because the optimal scheduling framework is thought to be numerous [28]. The need for more thorough investigations into the computational complexity and practical implementation difficulties associated with optimizing PMU placement strategies, especially in large-scale power systems with zero injection constraints, is a research gap in the current literature on PMU placement approaches.

The primary target of the present work is to examine the importance of economically placing distribution-stage phasor measurements while considering zero injections to improve system observability.

The key offerings of this study are enumerated as follows: 1) Developed a method to choose the ideal PMU position for radial distribution systems taking ZI buses into consideration.

2) This methodology examines voltage estimation using PMU measurements while considering zero injections. The ZI buses lower the system’s necessary PMU count, making the network fully visible and cost-effective.

3)The network topology is the primary consideration for determining the PMU positions. Any installation of voltage-regulating equipment, including distributed generation units, has no impact on these positions.

4) This approach can be applied to radial feeder structures regardless of their size or the diversity of X/R ratios.

## 2. Optimal PMU placement on full observability while considering regular system operation

Observability of a bus refers to the ability to measure the state variables (e.g., voltage and phase angle) at a particular bus in an electrical power system. PMU technology can be used to increase the observability of a bus. Increasing the observability of PMU buses is crucial for ensuring the stability, reliability, and security of power systems.

A zero-injection bus is a bus in an electric power system that has no net power injection or consumption. This means that the sum of the active power and reactive power injections at the bus is equal to zero. In other words, the power flowing into the ZI buses from connected transmission lines is equal to the power flowing out of it. The concept of ZI buses is significant in power system analysis and control because they can be used as reference points for voltage and phase angle measurements. Since there is no power injection or consumption at a ZI buses, the voltage magnitude and phase angle at the bus are determined only by the power injections at neighbouring buses. Therefore, by measuring the voltage and phase angle at a ZI buses, the voltage and phase angle at neighbouring buses can be estimated.

### 2.1 Criteria for a bus to be observable

**Fig 1:**
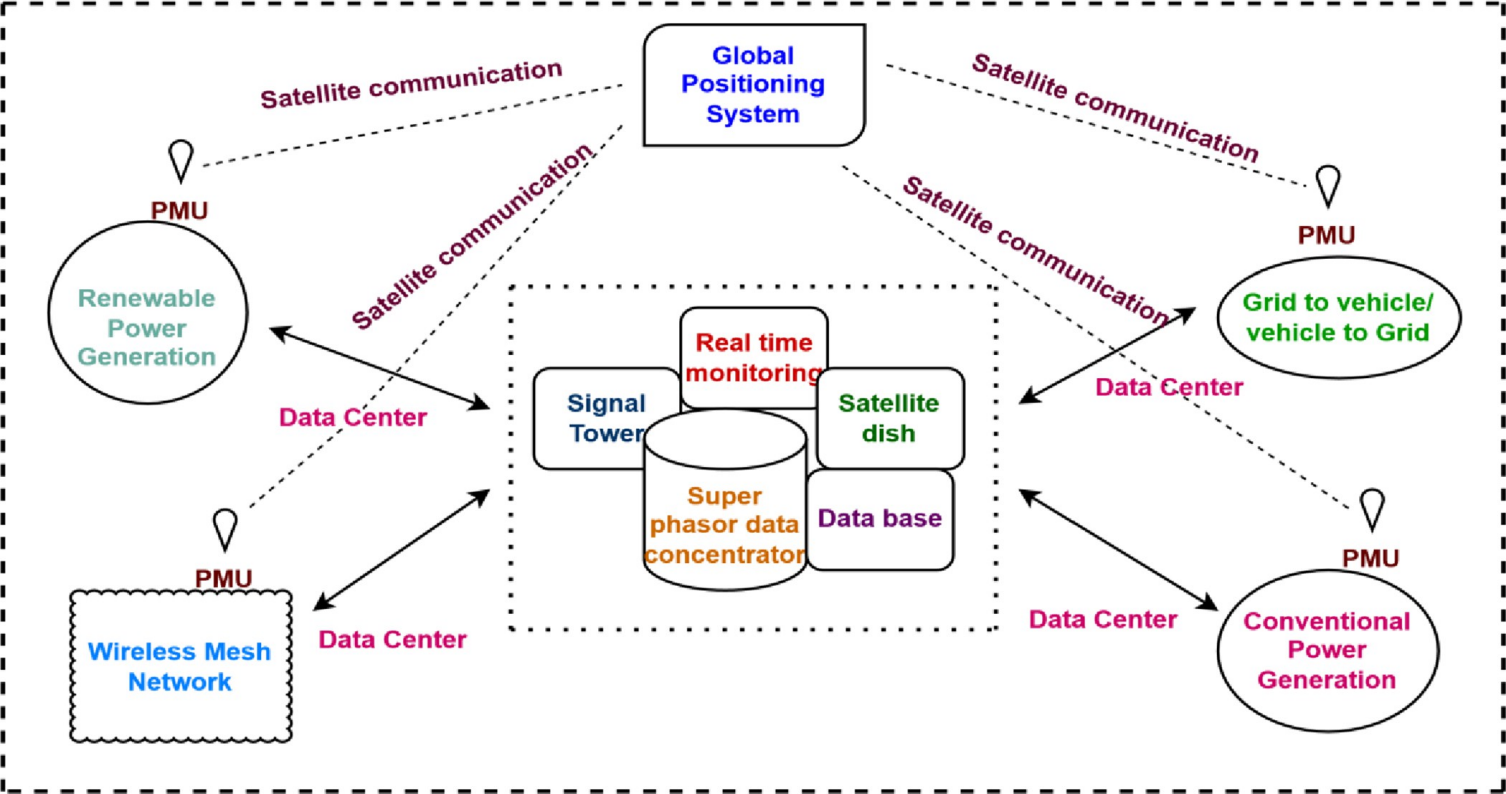
Wide area smart grid model with PMUs.

In the observability analysis of power systems, topological and numerical observability are the two main methods that are frequently used. From a mathematical perspective, a system is observable when the rank of the related Jacobian matrix or measurement gain matrix equals the system’s unknown states. However, in the case of enormous systems, the numerical observability assessment is often seen as a cognitive expense. It fails to identify the genuine zero diagonal members of the matrix due to potential rounding mistakes. As a result, most articles have used topological methodologies to confirm the system’s complete observability [29]. The wide area Smart Grid model with PMUs are plotted in Fig 1.

### 2.2 Criteria to monitor the buses in any power system

1. The phasors voltage/current of the associated bus can be evaluated by a PMU when installed on that bus.
2. A PMU deployed at bus permits for surveillance of that bus and all neighbouring buses. While PMU can directly measure the voltage magnitude of the node it is equipped with, KCL is used to ascertain the voltage phasor of the connecting node [30].
3. When one bus cannot be observed in the set of ZI buses and all associated buses, its values are calculated using kirchhoff’s current law (KCL). Additionally, KCL can be used to determine the value of a set of connected buses that conform to a ZI buses.

#### 2.2.1 Limitations for ILP when no zero-injection bus is considered


$$\min\;\sum_{k=1}^{N}q_{k}$$ (1)

SubjecttoG=PQ≥L (2)

$$Q={\left[q_{1,}q_{2,\ldots \ldots \ldots \ldots \ldots \ldots .}q_{n}\right]}^{T}$$ (3)

$$q_{k}\in \{0,1\},\;k=1,2,\ldots \ldots \ldots .,Z$$ (4)

*Z* is the total number of nodes in the system

QisthecolumnmatrixofPMUposition (5)

$$q_{k}=\left\{ \begin{array}{c} 1,\;if\;PMU\;is\;needed\;in\;bus\;k\\ 0,\;otherwise \end{array}\right.$$ (6)

The matrix element of *P* are as follows,

$$P(i,j)=\left\{ \begin{array}{c} 1,\;if\;buses\;i\;and\;j\;are\;connected\\ 1,\;i=j\\ 0,\;if\;buses\;i\;and\;j\;are\;not\;connected \end{array}\right.$$ (7)

$$L={\left[1\;1\;1\ldots \ldots \ldots \ldots .1\right]}^{T}$$ (8)

$$g_{i}=\sum_{j=1}^{N}\;P_{ij}\;q_{j}\geq 1;\;where\;i=1,2,\ldots \ldots ..,N$$ (9)

The observability of the *i*_*th*_ bus is determined by *g*_*i*_, which is determined by the connection matrix *P*. *L* is the column matrix, which shows the least number of PMUs to be installed to make the set of buses observable [8]. A comparison between the PMU placement techniques considering zero injection constraints is given in Table 1.

**Table 1:** Exploration of PMU placement techniques considering zero injection constraints.

Reference	Methodology	Key Features	Focus	Contributions	Drawbacks/Limitations
[2]	Modified Newton method and fast decoupled state estimation	Computational efficiency, numerical stability, zero injection constraints	State estimation	Improved state estimation methods with minor code modifications	Limited discussion on practical implementation challenges
[11]	Unified PMU placement model with ZI observation depth	Reliability, reduced PMUs, observability	PMU placement	Optimal PMU placement with reliable ZI observations	May require complex integer linear programming (ILP) solving for large systems
[23]	ILP model for PMU placement considering ZIBs and CMs	Observability, contingencies	PMU placement	Superior ILP model for PMU placement under contingencies	Computational complexity increases with network size
[25]	Security-oriented PMU placement with ZIBs	Network observability, redundancy, security	PMU placement	Innovative approach for secure PMU placement	Potential complexity in modelling security concepts
[11]	Unified PMU placement model with ZI reliability	Reliability, reduced ZI utilization	PMU placement	Globally optimal PMU placement with improved ZI observations	ILP-based methods may require significant computation
[12]	Modeling ZIBs for efficient PMU placement	Observability constraints, efficiency	PMU placement	Efficient PMU placement based on ZIB modelling	Complexity in defining and implementing observability constraints
[34]	ILP model with weighted redundancy levels	Measurement redundancy, reduced PMUs	PMU placement	Improved measurement redundancy distribution	Optimization may become challenging for large-scale systems
[35]	Weak bus identification for PMU placement	Weak bus identification, observability	PMU placement	Effective PMU placement considering weak buses	Effectiveness depends on the accuracy of weak bus identification methods

#### 2.2.2 Variation in constraints when ZIBs are taken into consideration

Certain adjustments must be made to the formulation of restrictions when a single ZIB or a collection of ZIBs is considered. Those adjustments are:

1. The *g*_*i*_ of a specific bus stays the same while not connected to any ZIB.
2. The buses linked to a single ZIB make up "*r*" members. These can be observed when the "*r*−1" members are visible.
3. Consequently, it is comprehended that,
$$\sum_{i=1}^{r}g_{i}\geq r-1$$ (10)
4. An "*r*" member set is created when there are "*s*" numbers of ZIBs, and they are all connected to certain buses. If ‘*r*−*s*’ members are observable, then these members are also observable.
$$\sum_{i=1}^{r}g_{i}\geq r-s$$ (11)
5. Buses should only appear in one ZIB when they are present in several ZIBs.
6. The new values of *P*_*new*_ and *L*_*new*_ are acquired when these rules have been instituted.

## 3. Methodology

### 3.1 Formulation for determining the best PMU location in systems with ZIBs

Two hypotheses are being considered if ZIBs are present.

#### 3.1.1 Case 1: A single ZI bus is present

The data file containing the line information for the individual bus systems is loaded. After that, the computation and display of the connection matrix relating to the line details are generated. If there are no ZI buses, the lower and upper bounds are set to 0 and 1 accordingly, L is given the value 1, and intcon is set as a series of numbers from 1 to the whole number of buses. Once the required assignments have been made, the arguments are passed to the intlinprog function, which returns the ideal PMU number and its locations explained in algorithm 1.

**Algorithm 1** Determine ZI bus-related information when a single ZI bus is present

**Require:** Input: ZI bus position

1: Create an empty array injection_bus Array

2: Set the first element of injection_bus Array to the input ZI bus

3: **for** each additional bus connected to the injection bus

  4: **do** find the bus

  5: Add it to injection_bus Array

6: **end for**

7: Create a separate array of non-injection connected buses for buses connected to the injection bus but not to any other injection buses

8: Modify the arrays to ensure they are the same size

9: Create a new connection matrix for buses with no injection, preserving original values

10: Create an array to track connections between buses and injection buses

11: Combine the elements of these matrices into a 1-dimensional array

12: Generate a column vector of ones for buses with no injection

13: Generate a vector r with a value of ‘*r*− 1’ for the injection bus

14: Combine the total injection bus locations and the connection matrix for no injection into a 1D array ‘P_new.’

15: Create L new by concatenating the column vector of ones for no injection and the one for injection buses

16: Repeat steps 3 to 4 using ‘P_new’ and ‘L_new’ as input values

#### 3.1.2 Case 2: Multiple ZIBs are present

When more than one ZIB is input, Case 1’s steps (a) through (c) are repeated for each ZIB until linking buses for all ZIBs are obtained. Iteratively displayed values are recorded in cells for each iteration that relate to the buses connected to each injection bus. The cell is subsequently transformed into a one-dimensional array containing each connecting injection bus. In addition to being sorted, this array is verified for bus duplication. The steps (d-g) are then repeated except for (g), which is altered so that the value of observability for the injection buses is changed to "*r*−*s*" explained in algorithm 2.

**Algorithm 2:** Determine Multiple ZI buses related bus information

 **Require:** Input: List of ZI bus positions

1: Create an empty array injection bus

2: Create an empty array connection Matrix

3: **for** each ZI bus in the list of ZI bus positions

4: **do** Set the current ZI bus as the input ZI bus

  5: Create an empty array current_ZI bus

  6: Set the first element of current_ZI bus to the current ZI bus

  7: **for** each additional bus connected to the current ZI bus

  8: **do** find the bus

  9: Add it to current_ZI bus array

10: **end for**

11: Add current ZI bus array to injection Bus array

12: Create a cell containing current_ZI bus array

13: **end for**

14: Transform the cell into a 1-dimensional array combined containing all connecting injection buses

15: Sort combined array

16: Verify and remove any duplicate buses from the combined array

17: Create a connection matrix for the buses in the combined array

18: Perform modifications on the connection Matrix

19: Change observability values for injection buses to ‘r-s’.

**Fig 2:**
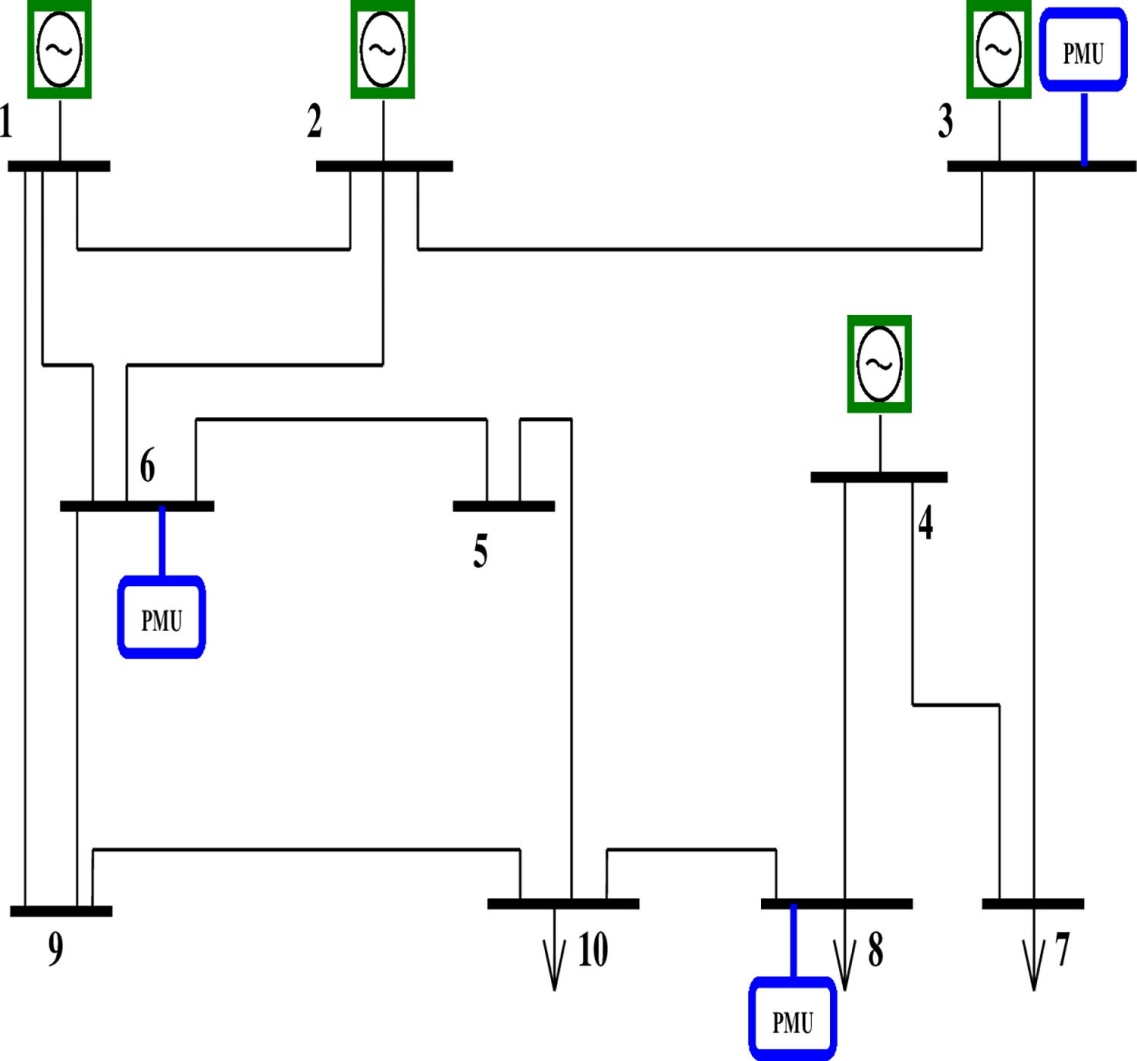
10 bus network without zero injections.

**Fig 3:**
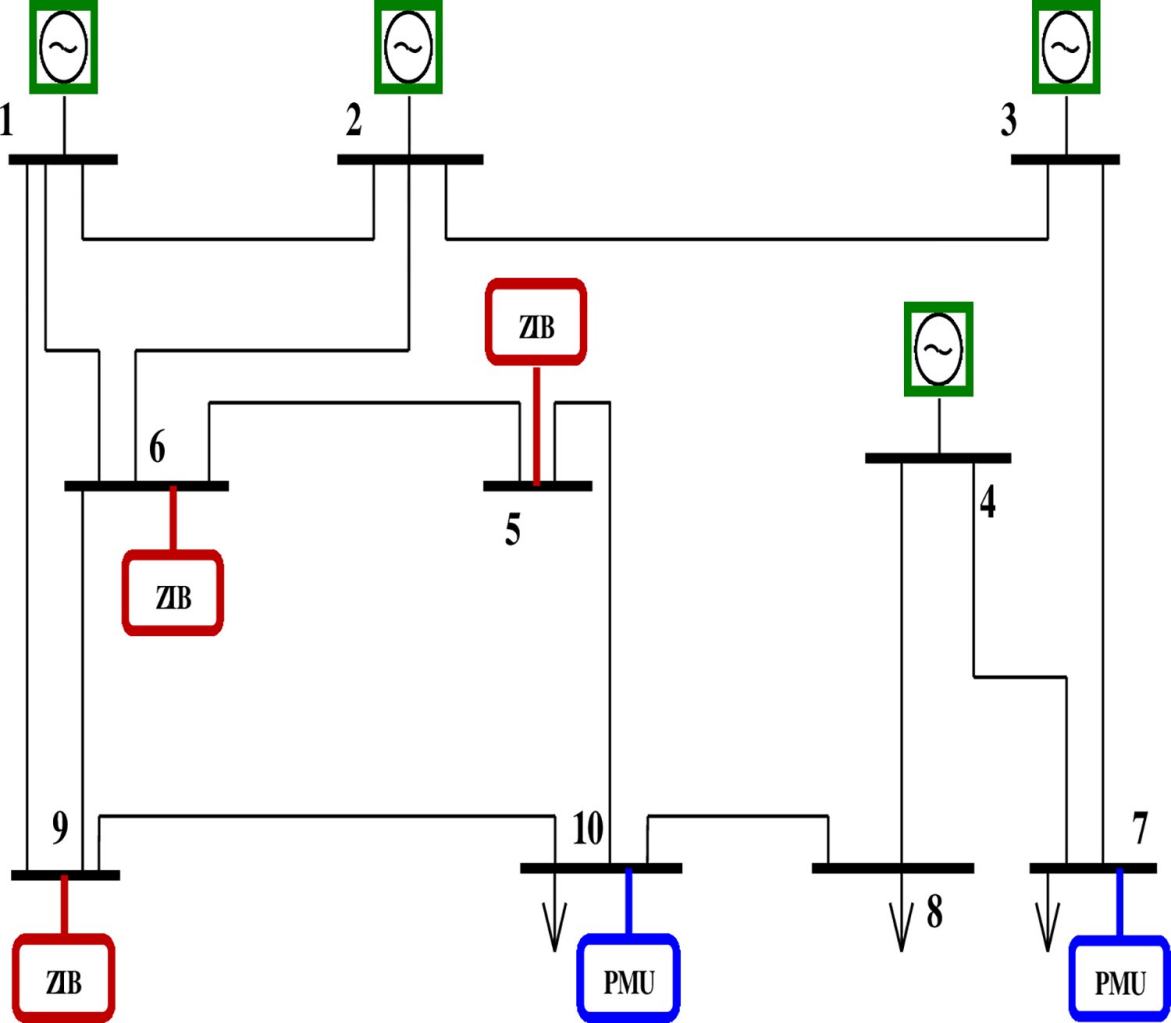
10 bus network with zero injections.

The above two cases are explained by considering a 10-bus system with zero injection (ZI) at positions 5, 6 & 9 as shown in Figs 2 and 3.

The initial connection matrix and the corresponding observability matrix are as follows,

$$P=\left[\begin{array}{c} 1\;1\;0\;0\;0\;1\;0\;0\;1\;0\\ 1\;1\;1\;0\;0\;1\;0\;0\;0\;0\\ 0\;1\;1\;0\;0\;0\;1\;0\;0\;0\\ 0\;0\;0\;1\;0\;0\;1\;1\;0\;0\\ 0\;0\;0\;0\;1\;1\;0\;0\;0\;1\\ 1\;1\;0\;0\;1\;1\;0\;0\;1\;0\\ 0\;0\;1\;1\;0\;0\;1\;0\;0\;0\\ 0\;0\;0\;1\;0\;0\;0\;1\;0\;1\\ 1\;0\;0\;0\;0\;1\;0\;0\;1\;1\\ 0\;0\;0\;0\;1\;0\;0\;1\;1\;1 \end{array}\right]$$ (12)

$$L=\left[\begin{array}{c} 1\\ 1\\ 1\\ 1\\ 1\\ 1\\ 1\\ 1\\ 1\\ 1 \end{array}\right]$$ (13)

With ZIBs at buses 5, 6 & 9

Buses connected to ZIB 5 are– 6 & 10

Buses connected to ZIB 6 are– 1, 2, 5 & 9

Buses connected to ZIB 9 are– 1, 6 & 10

The buses that do not have any injections are 3, 4, 7 & 8.


$$Hence,\;\begin{array}{c} g_{3}=q_{2}+q_{3}+q_{7}\geq 1\\ g_{4}=q_{4}+q_{7}+q_{8}\geq 1\\ g_{7}=q_{3}+q_{4}+q_{7}\geq 1\\ g_{8}=q_{4}+q_{8}+q_{10}\geq 1 \end{array}\}$$ (14)

$$\begin{array}{c} g_{6}+g_{10}=q_{1}+q_{2}+2q_{5}+q_{6}+q_{8}+{2q}_{9}+q_{10}\geq 1\\ g_{1}+g_{2}+g_{5}+g_{9}={3q}_{1}+{2q}_{2}+q_{3}+q_{5}+{4q}_{6}+{2q}_{9}+{2q}_{10}\geq 2\\ g_{7}+g_{3}+g_{3}={2q}_{1}+2q_{2}+{2q}_{5}+{2q}_{6}+q_{8}+{3q}_{9}+q_{10}\geq 2 \end{array}\}$$ (15)

Buses with injection are 1, 2, 5, 6, 9 & 10

No: of elements in the injection array set (r) = 6

No: of ZI buses = 3

Therefore (*r*−*s*) = 3 which means 3 buses of the injection buses need to be observable.

Hence,

$$g_{1}+g_{2}+g_{5}+g_{6}+g_{9}+g_{10}={4q}_{1}+3q_{2}+q_{3}+{3q}_{5}+{5q}_{6}+q_{8}+{4q}_{9}+{3q}_{10}\geq 3$$ (16)

$$P_{new}=\left[\begin{array}{c} 0\;1\;1\;0\;0\;0\;1\;0\;0\;0\\ 0\;0\;0\;1\;0\;0\;1\;1\;0\;0\\ 0\;0\;1\;1\;0\;0\;1\;0\;0\;0\\ 0\;0\;0\;1\;0\;0\;0\;1\;0\;1\\ 4\;3\;1\;0\;3\;5\;0\;1\;4\;3 \end{array}\right]$$ (17)

$$L_{new}=\left[\begin{array}{c} 1\\ 1\\ 1\\ 1\\ 3 \end{array}\right]$$ (18)

With injection the PMU numbers and PMU locations are determined as follows,

Number of PMUs = 2

The positions of PMUs are Q = [0 0 0 0 0 0 1 0 0 1]

Therefore, PMUs are to be positioned in buses 7 and 10. The total PMUs needed for the 10 bus network without considering ZI buses were three and were located in positions 3, 6, and 8.

#### 3.1.3 Flowchart of OPP with and without ZIB

**Fig 4:**
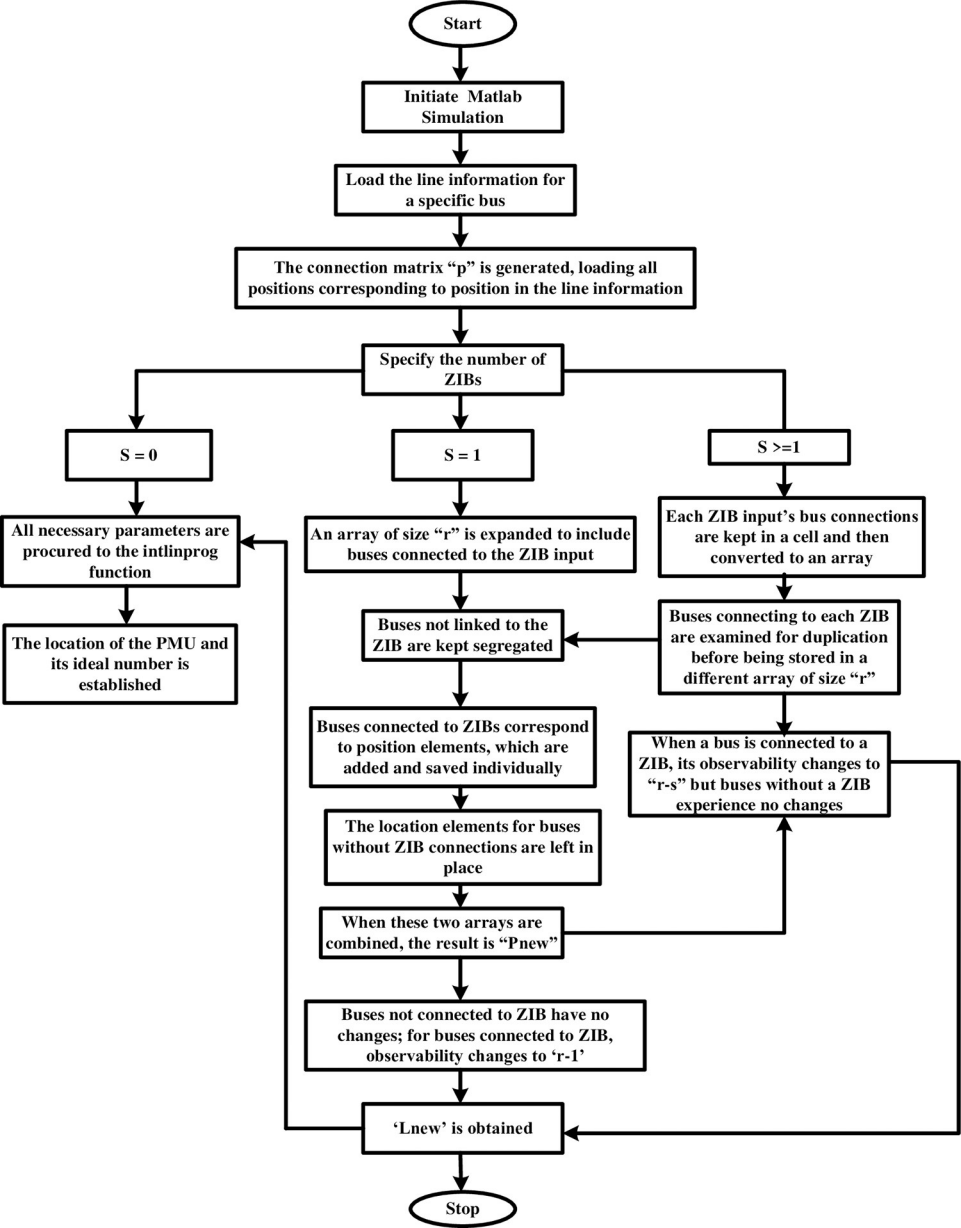
Flowchart of OPP with and without ZIB.

The flow chart of the OPP with and without ZIB is depicted in Fig 4.

### 3.2 Formulation for the voltage estimation using the best PMU location in systems with ZI buses

A generic algorithm is used to evaluate the voltages and currents of the network using PMU-associated nodes. The ZI buses bus numbers are input to get the new connection matrix. The bus numbers at the end of each adjacent chain and the focal chain of buses make up the enbus vector. First, a voltage of one p.u. (per unit) is assumed on each bus [31–33]. The line currents are determined by using the enbus vector and KCL for the remaining lines of end buses. Kirchhoff’s voltage law (KVL) is used to ascertain the node voltages by working back from the last node to the first node and accessing the line currents from the preceding. This process was repeated using the earlier bus voltages to obtain the line currents. The sequence will be terminated if the variation between the voltage magnitudes is exceedingly tiny. The voltage and line current vectors are utilized to evaluate the voltages and currents at various PMUs by identifying the line matrix with all bus interconnections [36, 37].

**Algorithm:** PMU framework for the voltage estimation considering Zero injection buses

        1: Start

        2: Load the bus specifics and their number in the network

        3: Store total no. of zero injection nodes (*Z*)

       4: Find the connection matrix; for *l = 1*: *total*_bus

    *connection_matrix(l*,*l) = 1*;

    *end*

        5: If *Z = = 0 (zero injection nodes are absent)*

        6: display the connection_matrix

         7: input lower & upper bound values

         8: Find the best possible PMU locations using intlinprog

        9: display the pmu_position

       10: If *Z ≠ 0 (non zero injection nodes)*

      11: *w* = input the *ZI* bus numbers

       12: Sort out the network with zero injections and find the injection matrix and new

      connection matrix with injections(connectionnewnoinj)

       13: display the connectionnewnoinj and repeat steps from 7 to 9

       14: Obtain the branch vector and PMU location.

       15: Get the real and reactive (*Px*, *Qx*) powers at each bus and line data (*Zx*).

16: Initialize **the** voltage of each bus as *Vx(l*,*1) = 1 p*.*u*.

       17: Form an enbus vector comprising end situated nodes; *enbus = Zeros(m*,*1)*.

18: Calculate line flows, accounting for end branches and remaining nodes. *Ix(m*,*1) = conj(abs(app_power)/(abs(Vx(m*,*1))))*.

       19: Use the line currents to estimate the new set of node voltages. *Vxnew = Vx(m*,*1)*.

20: Execute another assessment of the line currents with the updated node voltages. *Ixnew = [l]*.

21: Continue looping until the difference between the voltages is less than the tolerance.

       22: Verify the parameters in the line current and node voltage vectors.

            *Vx*(pmu_node(k)), *Ix*(pmu_node(k)-1) of the PMU measurement.

       23: Print the results of node voltage and line current vectors.

       24: Stop.

## 4. Results & discussions

This section explains the findings from the best PMU placement with and without ZI buses and assesses the voltage profile using PMUs. The simulations are executed on the MATLAB platform.

### 4.1 Optimal PMU locations

For full observability of the distribution test feeders, an integer linear programming technique is utilized in the simulation with a minimum number of PMUs with and without ZI buses. Table 2 shows the complete observability of these test feeders, and the requisite PMU counts and placements for the case studies on the modified IEEE 18-bus, 33-bus, and 69-bus networks. When ZI buses are considered in the design of PMU placement, the necessary number of PMUs is significantly reduced without affecting the system’s observability [31–35].

**Table 2:** IEEE radial bus networks’ optimal PMU bus placements with and without ZI buses.

IEEE Test Systems	PMUs count without ZI buses	Positions of PMU	No. of ZI buses	ZI buses location	No. of PMUs with ZI buses	Positions of PMU
18	7	1,4,7, 10, 13, 16, 18	3	2,3,7	6	3, 4, 9, 13, 16, 18
33	11	2,5,8,11,14,17,21,24,27,30,33	2	6, 27	10	2, 5, 8, 11, 14, 17, 21, 24, 29, 32
69	24	2,6,9,14,17,20,23,26,29,32,35,37,40,43,46,47,50,52,55,58,61,64,66,68	20	2, 3, 4, 5, 15, 19. 23, 25, 30, 31, 32, 38, 42, 44, 47, 56, 57, 58,60, 63	16	3 4 6 14 17 20 26 34 41 45 49 51 54 64 66 68

### 4.2 Voltage profile estimation of IEEE bus systems using PMUs

The 18-bus data is provided in [35], whereas data for 33 and 69 buses are provided in [36].

#### 4.2.1 18 bus results

**Fig 5:**
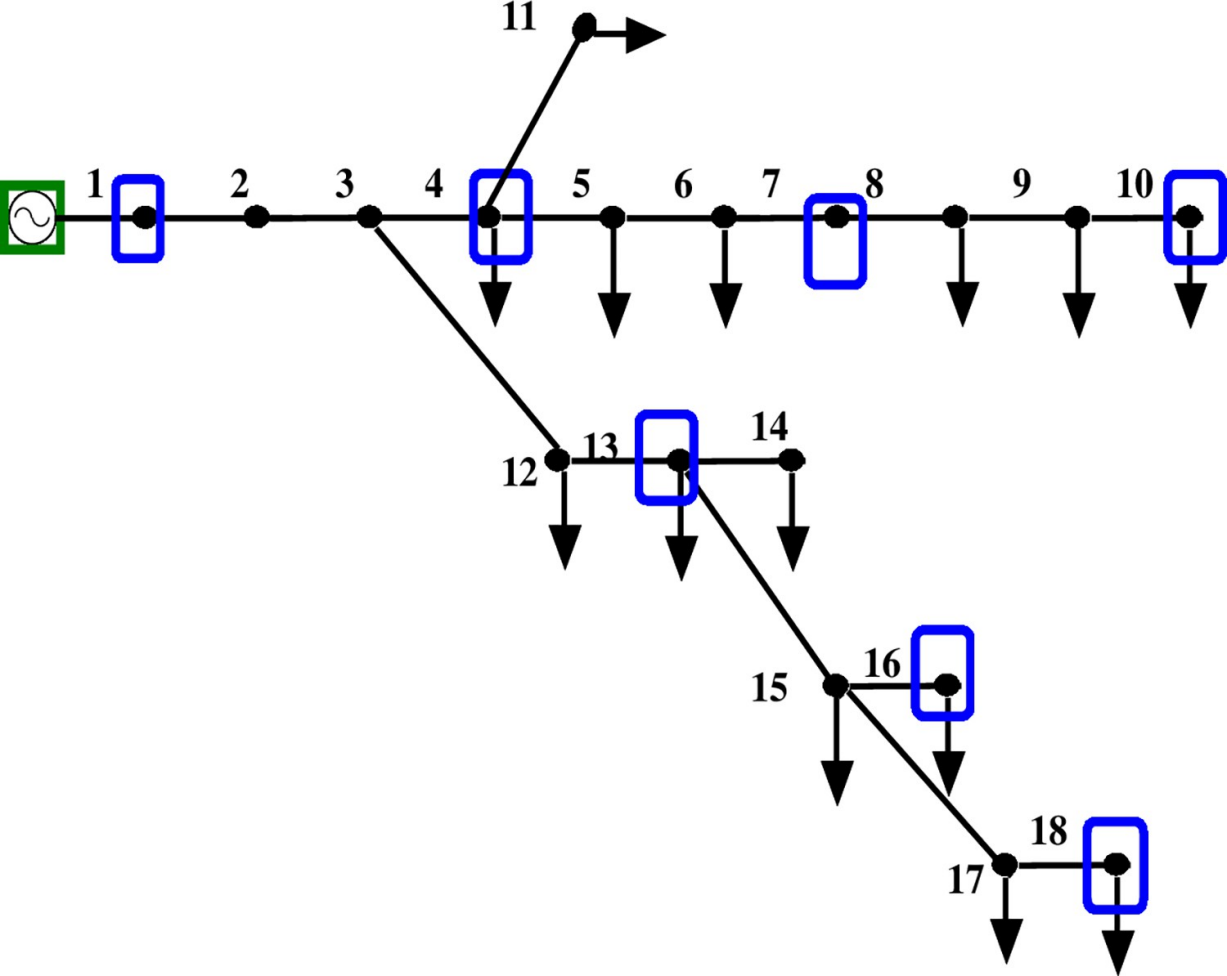
ZIB-free modified IEEE 18 bus network.

**Fig 6:**
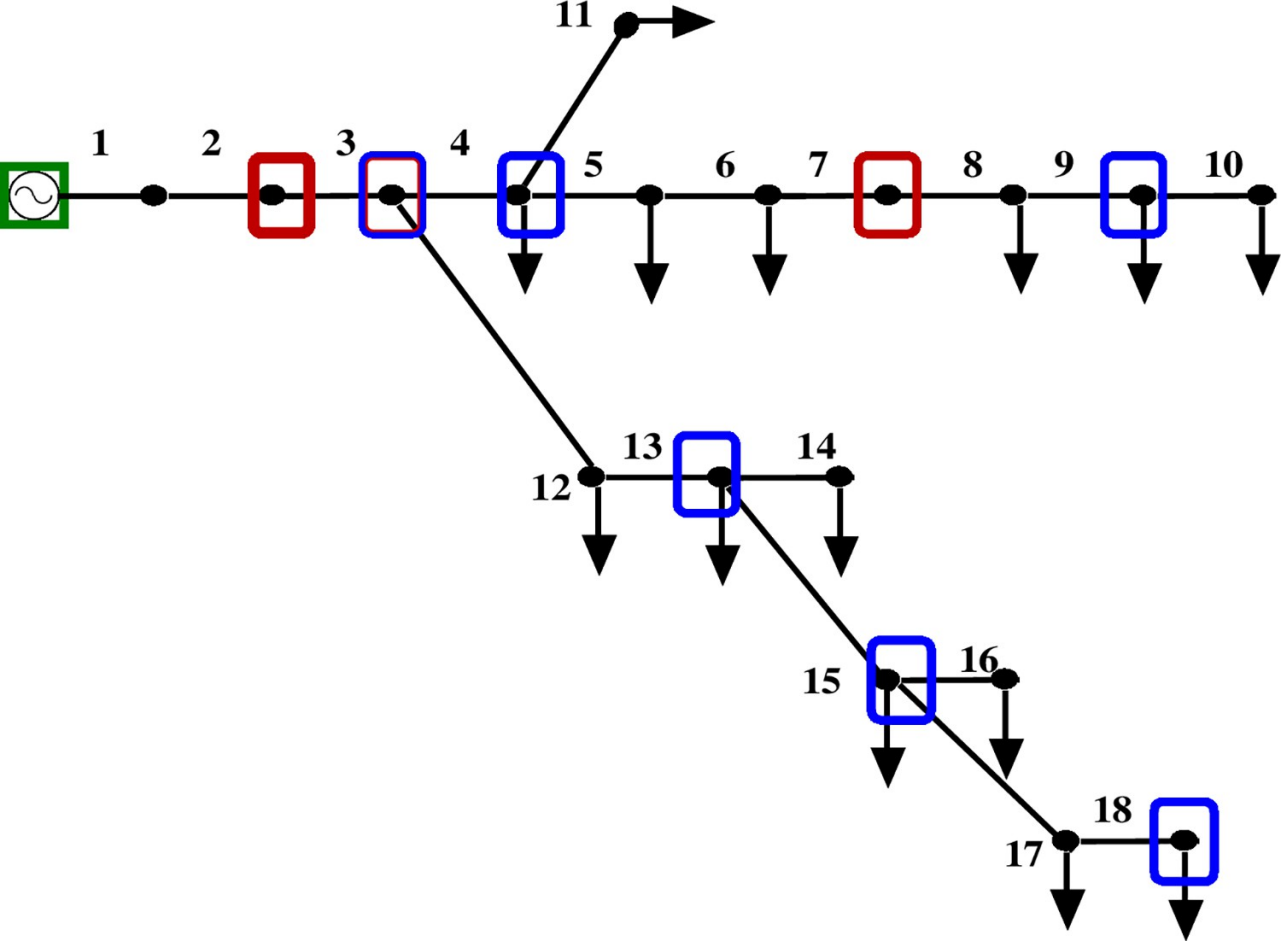
ZIB-enabled modified IEEE 18 bus network.

Figs 5 and 6 depict the 18-bus radial distribution network with PMUs at designated nodes, both with and without ZI buses.

**Fig 7:**
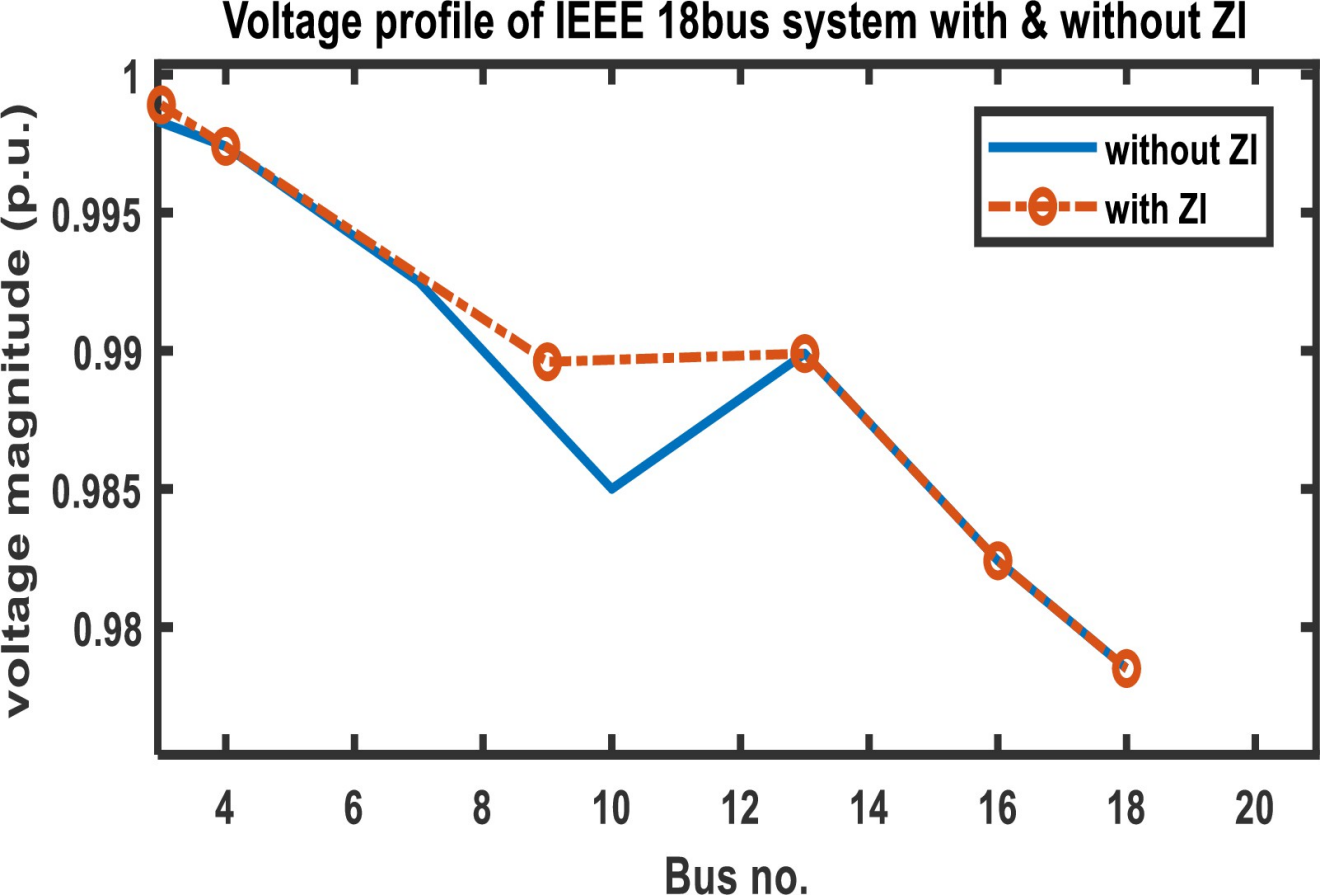
Modified IEEE 18 bus system voltage profile with and without ZI buses.

Table 3 displays the results of comparing the voltage magnitude of an 18-bus system with and without ZI buses. The comparison shows that using ZI buses minimizes the number of PMUs deployed in a bus network while retaining its observability. Additionally, Fig 7 demonstrates that the usage of ZI buses has no appreciable impact on the voltage profile of the network.

**Table 3:** Voltage magnitudes of modified IEEE 18 bus system at various PMU locations.

Without ZIB		With ZIB	
PMU location	Voltage mag. (p.u.)	PMU location	Voltage mag. (p.u.)
1	1	3	0.9989
4	0.9974	4	0.9974
7	0.9925	9	0.9896
10	0.9850	13	0.9899
13	0.9899	16	0.9824
16	0.9824	18	0.9785
18	0.9785		

#### 4.2.2 33 bus results

**Fig 8:**
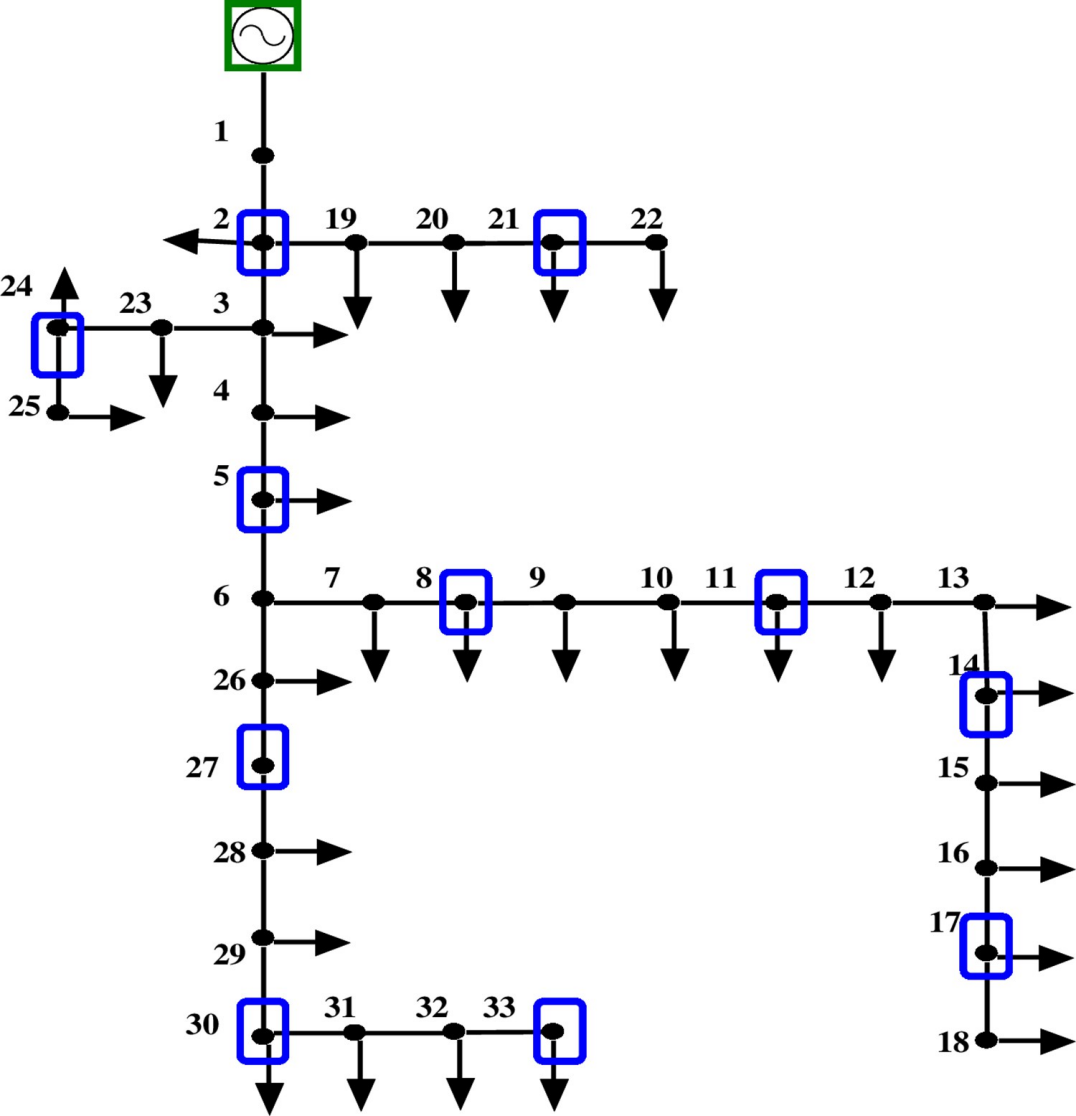
IEEE 33 bus network without ZI buses.

**Fig 9:**
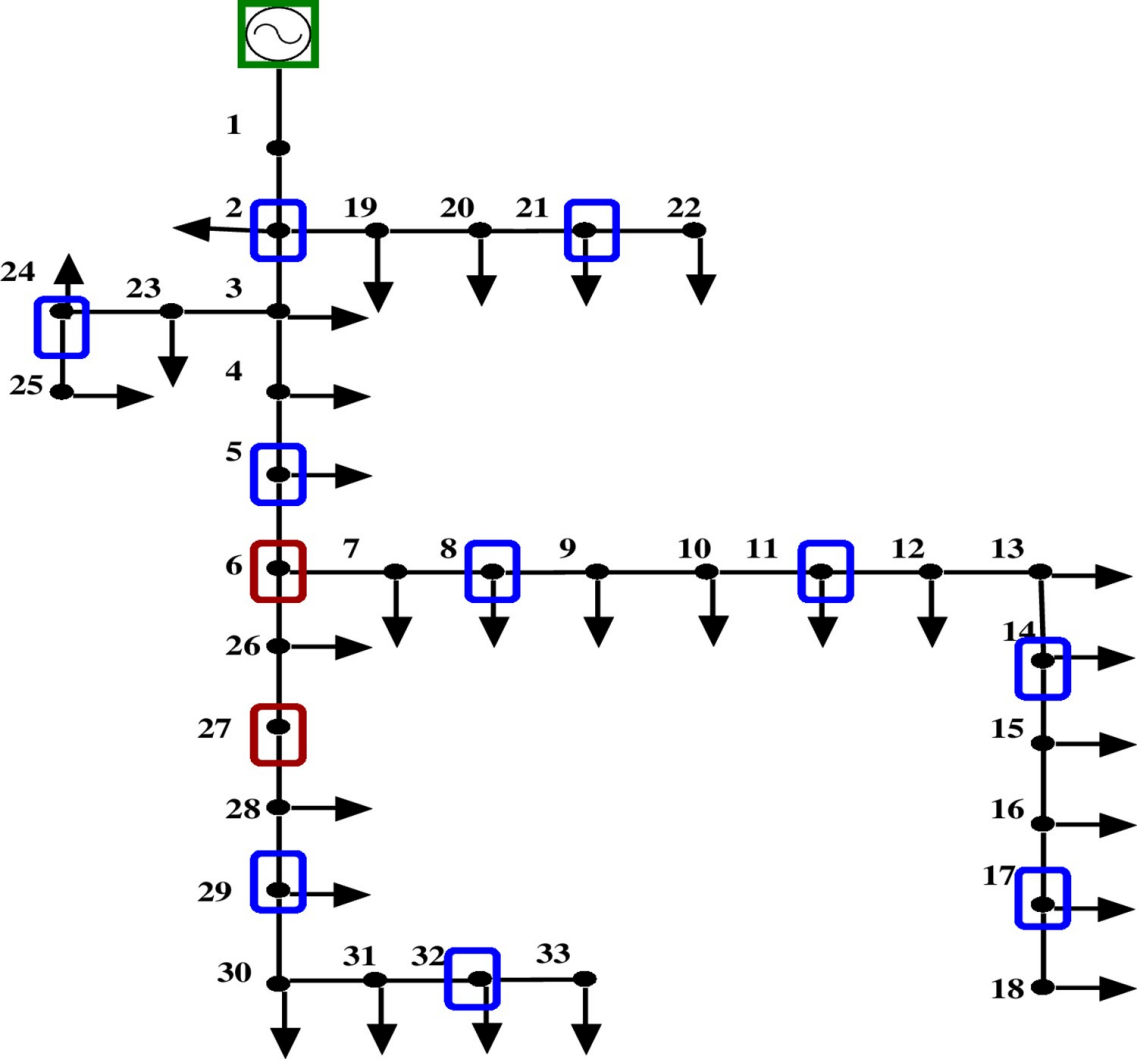
IEEE 33 bus network with ZI buses.

Figs 8 and 9 depict the 33-bus radial distribution network with PMUs at designated nodes, both with and without ZIs.

**Fig 10:**
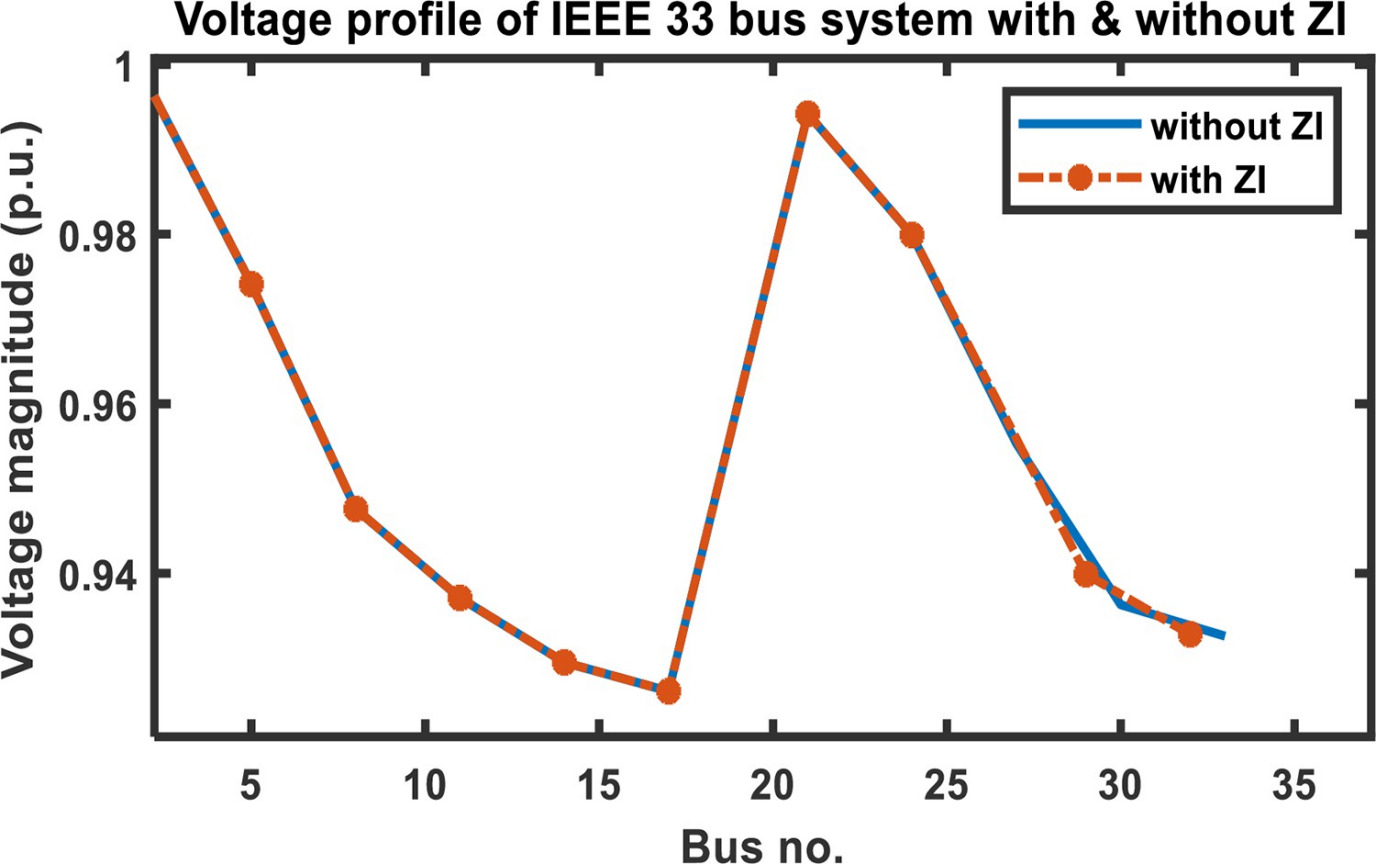
IEEE 33 bus system voltage profile with and without ZI.

The results from evaluating the voltage magnitude of a 33-bus system with and without ZIs are shown in Table 4. According to the comparison, employing ZI buses reduces the quantity of PMUs used in a bus network while maintaining its observability. Additionally, Fig 10 shows that the voltage profile of the network is unaffected significantly using ZI buses.

**Table 4:** Voltage magnitudes of IEEE 33 bus system at various PMU locations.

Without ZI buses		With ZI buses	
PMU position	Voltage mag. (p.u.)	PMU position	Voltage mag. (p.u.)
2	0.9979	2	0.9979
5	0.9741	5	0.9741
8	0.9476	8	0.9476
11	0.9371	11	0.9371
14	0.9295	14	0.9295
17	0.9261	17	0.9261
21	0.9942	21	0.9942
24	0.9799	24	0.9799
27	0.9553	29	0.9399
30	0.9363	32	0.9328
33	0.9326		

#### 4.2.3 69 bus results

**Fig 11:**
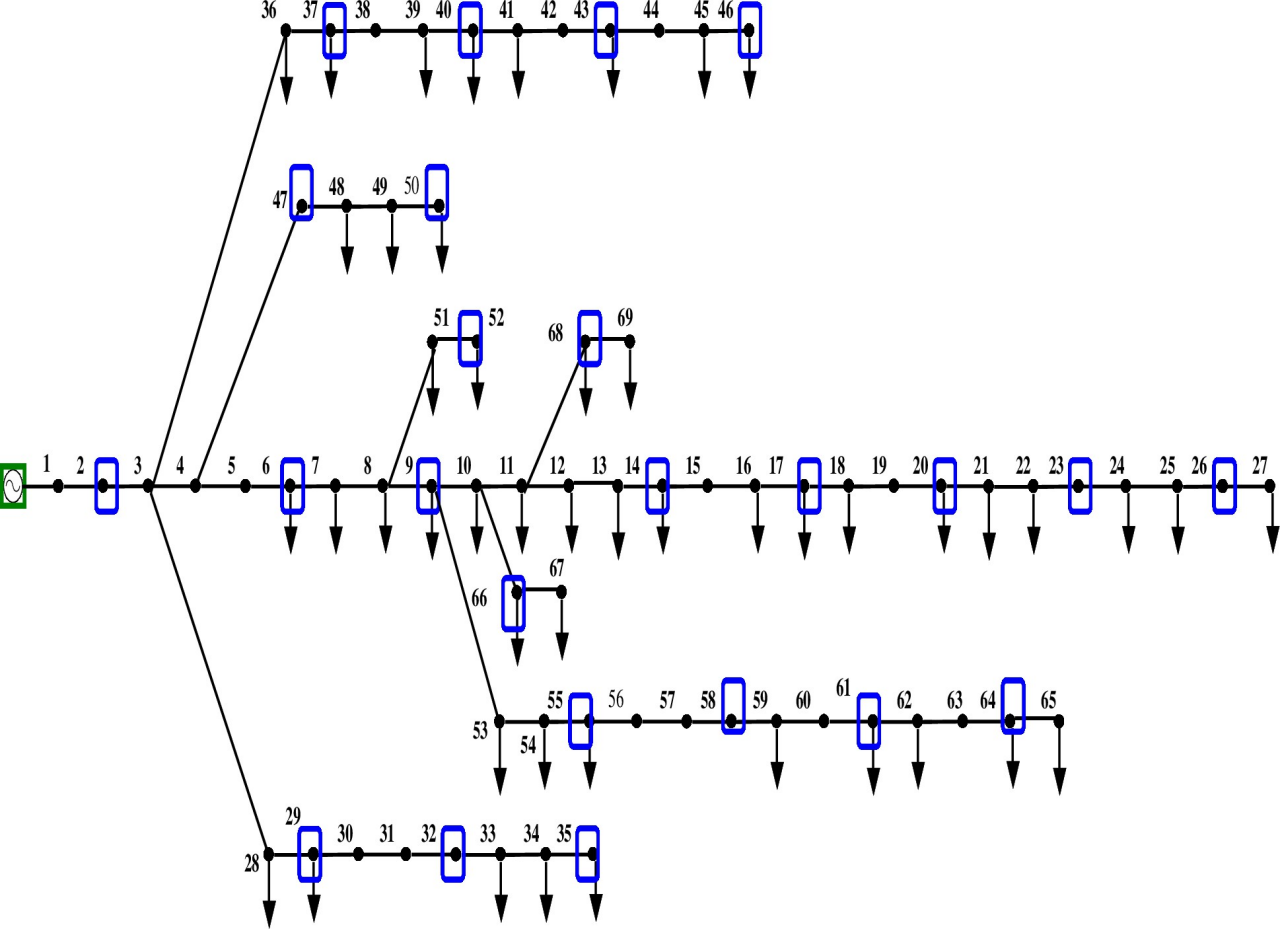
IEEE 69 bus network without ZI buses.

**Fig 12:**
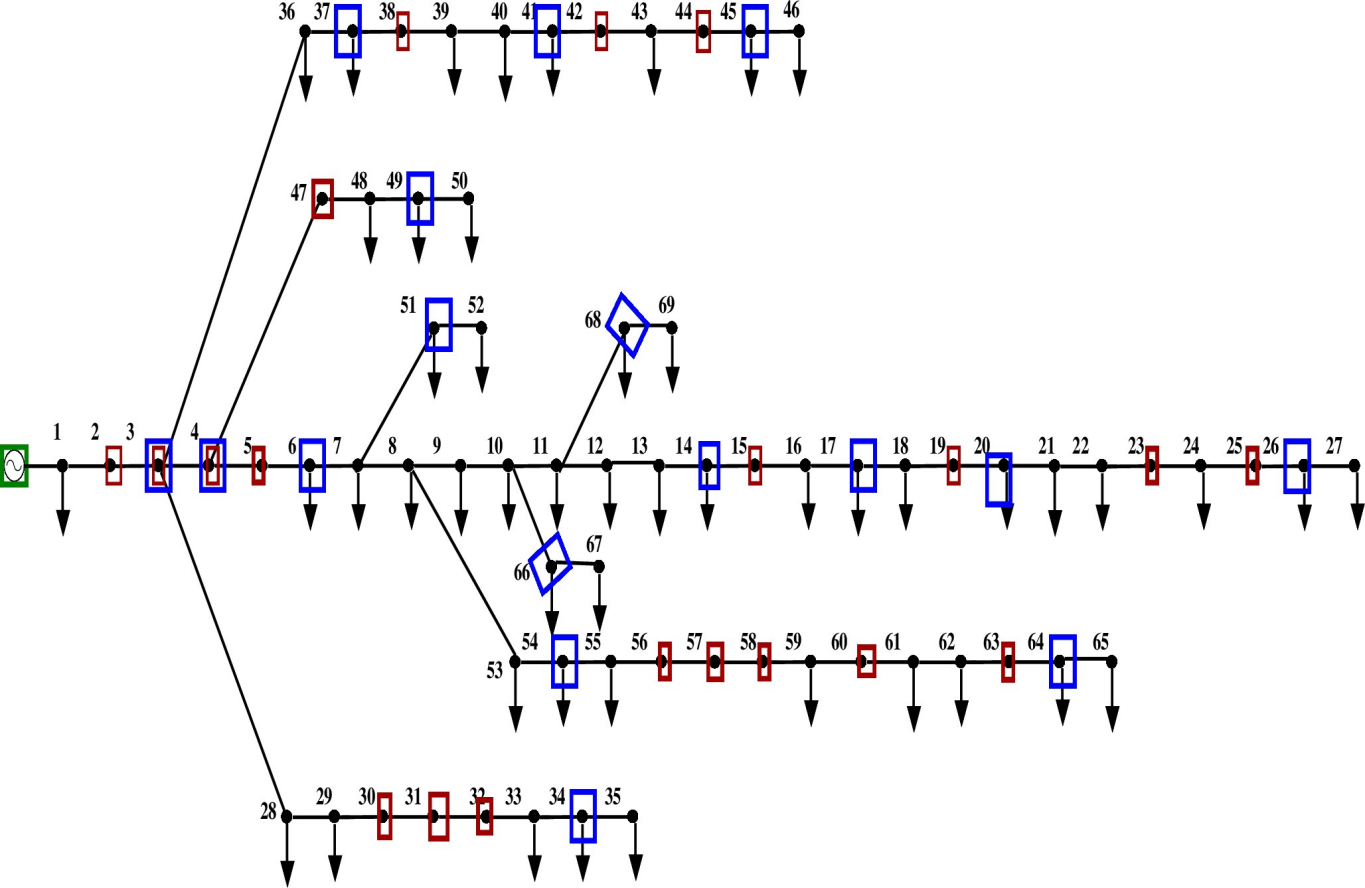
IEEE 69 bus network with ZI buses.

Figs 11 and 12 depict the 69-bus radial distribution network with PMUs at designated nodes, both with and without ZI.

**Fig 13:**
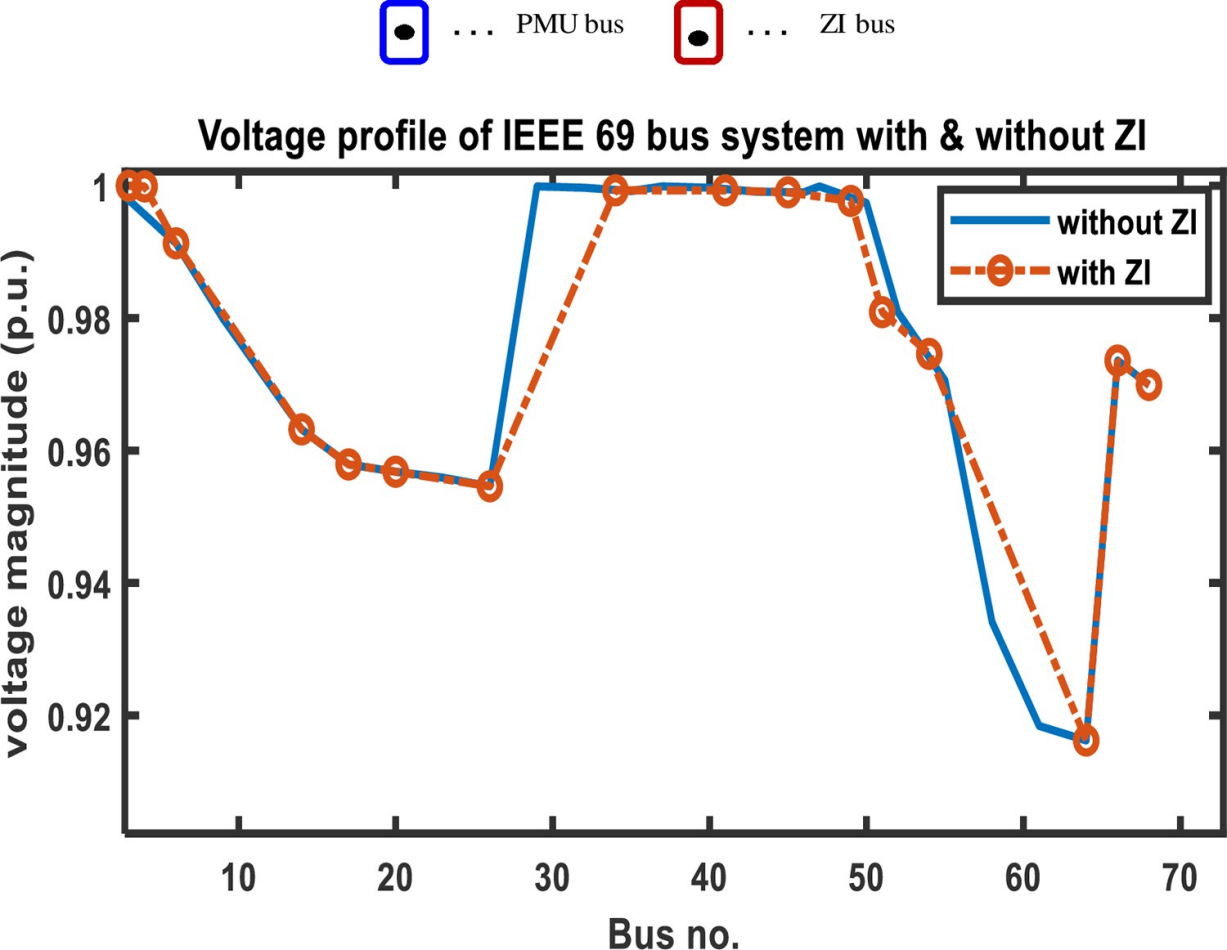
IEEE 69 bus system voltage profile with and without ZIs.

The findings from comparing the voltage magnitude of a 69-bus system with and without ZIs are shown in Table 5. This comparison shows that using ZI buses decreases the number of PMUs employed in a bus network while keeping it observable. Fig 13 further shows that the voltage profile of the system is unaffected significantly using ZI buses.

**Table 5:** Voltage magnitudes of IEEE 69 bus system at various PMU locations.

Without ZI buses		With ZI buses	
PMU locations	Voltage mag. (p.u.)	PMU location	Voltage mag. (p.u.)
2	1	3	1
6	0.9913	4	0.9999
9	0.9799	6	0.9913
14	0.9632	14	0.9632
17	0.9579	17	0.9579
20	0.9568	20	0.9568
23	0.9559	26	0.9546
26	0.9546	34	0.9993
29	0.9999	41	0.9993
32	0.9997	45	0.999
35	0.9992	49	0.9977
37	0.9999	51	0.9809
40	0.9997	54	0.9746
43	0.9991	64	0.9162
46	0.9990	66	0.9736
47	0.9999	68	0.9699
50	0.9974		
52	0.9809		
55	0.9706		
58	0.9341		
61	0.9184		
64	0.9162		
66	0.9736		
68	0.9699		

## 5. Conclusion

The aim of this study is to simplify the process of selecting the best locations for PMUs in different configurations of distribution networks. The study proposes two strategies for this purpose. The first strategy aims to identify the best locations for PMUs in the distribution network with and without ZI buses, which are zero injection buses where neither a load nor a generator is connected. The second strategy involves assessing the voltage profile of a radial distribution feeder using PMU technology under the conditions mentioned above. To achieve this objective, the study proposes leveraging ILP to install the required number of PMUs at different locations and operate the network normally. Once the installation is completed, ZI buses in the network are considered to reduce the number of PMUs required. The results of the study show that using ZI buses has no discernible effect on the voltage profile of the system, which demonstrates the effectiveness of this strategy. Overall, this study provides valuable insights into the selection of optimal PMU locations in distribution networks, which can help improve the accuracy and efficiency of power system monitoring and control. The use of ILP and PMU technology can simplify the process of selecting PMU locations and reduce the cost of installation and operation, making it more feasible to implement PMUs in distribution networks.
